# ANGPTL4 influences the therapeutic response of patients with neovascular age-related macular degeneration by promoting choroidal neovascularization

**DOI:** 10.1172/jci.insight.157896

**Published:** 2022-07-08

**Authors:** Yu Qin, Aumreetam Dinabandhu, Xuan Cao, Jaron Castillo Sanchez, Kathleen Jee, Murilo Rodrigues, Chuanyu Guo, Jing Zhang, Jordan Vancel, Deepak Menon, Noore-Sabah Khan, Tao Ma, Stephany Y. Tzeng, Yassine Daoud, Jordan J. Green, Gregg L. Semenza, Silvia Montaner, Akrit Sodhi

**Affiliations:** 1 Wilmer Eye Institute, Johns Hopkins University School of Medicine, Baltimore, Maryland, USA.; 2 Department of Ophthalmology, the Fourth Affiliated Hospital of China Medical University, Eye Hospital of China Medical University, Key Lens Research Laboratory of Liaoning Province, Shenyang, China.; 3 Department of Oncology and Diagnostic Sciences, School of Dentistry, Greenebaum Comprehensive Cancer Center, University of Maryland, Baltimore, Maryland, USA.; 4 State Key Laboratory of Ophthalmology, Clinical Research Center, Zhongshan Ophthalmic Center, Sun Yat-Sen University, Guangzhou, China.; 5 Department of Biomedical Engineering, Institute for NanoBioTechnology, and; 6 Translational Tissue Engineering Center, Johns Hopkins University School of Medicine, Baltimore, Maryland, USA.; 7 Department of Genetic Medicine,; 8 Department of Pediatrics,; 9 Department of Medicine,; 10 Department of Oncology,; 11 Department of Radiation Oncology, and; 12 Department of Biological Chemistry, Johns Hopkins University School of Medicine, Baltimore, Maryland, USA.

**Keywords:** Ophthalmology, Clinical practice, Molecular diagnosis, Molecular pathology

## Abstract

Most patients with neovascular age-related macular degeneration (nvAMD), the leading cause of severe vision loss in elderly US citizens, respond inadequately to current therapies targeting a single angiogenic mediator, vascular endothelial growth factor (VEGF). Here, we report that aqueous fluid levels of a second vasoactive mediator, angiopoietin-like 4 (ANGPTL4), can help predict the response of patients with nvAMD to anti-VEGF therapies. ANGPTL4 expression was higher in patients who required monthly treatment with anti-VEGF therapies compared with patients who could be effectively treated with less-frequent injections. We further demonstrate that ANGPTL4 acts synergistically with VEGF to promote the growth and leakage of choroidal neovascular (CNV) lesions in mice. Targeting ANGPTL4 expression was as effective as targeting VEGF expression for treating CNV in mice, while simultaneously targeting both was more effective than targeting either factor alone. To help translate these findings to patients, we used a soluble receptor that binds to both VEGF and ANGPTL4 and effectively inhibited the development of CNV lesions in mice. Our findings provide an assay that can help predict the response of patients with nvAMD to anti-VEGF monotherapy and suggest that therapies targeting both ANGPTL4 and VEGF will be a more effective approach for the treatment of this blinding disease.

## Introduction

Neovascular age-related macular degeneration (nvAMD), or “wet” AMD, is a leading cause of severe vision loss in elderly US citizens (1). The growth of abnormal leaky blood vessels (i.e., choroidal neovascularization [CNV]) in these patients can lead to rapid and often irreversible vision loss (2). The recent introduction of therapies targeting vascular endothelial growth factor (VEGF), a potent endothelial mitogen and permeability factor, has had a remarkable impact on patients with nvAMD who previously suffered vision loss from edema, bleeding, and scarring caused by CNV (3). However, anti-VEGF therapies are administered every 1–2 months indefinitely, raising concerns about the substantial economic and social burden of frequent clinic visits for elderly patients who often require assistance for transportation and mobility (4). This is particularly relevant given emerging ethical issues about the distribution of “cost-effective” versus “most effective” anti-VEGF therapies among patients with ocular neovascular disease (5). There are also concerns about ocular risks — and concerns based on preclinical data about the safety — of frequent, chronic intraocular injections of anti-VEGF therapies (6, 7). This has prompted investigators to explore alternative approaches that refine initial treatment protocols without sacrificing the visual acuity benefits observed with monthly or bimonthly treatment.

In this regard, we recently described a hybrid of the treat-and-extend (TAE) and pro re nata (PRN) protocols, which we designated TAE, pause, and monitor (TEP/M), in which patients initially undergo 3 consecutive monthly injections with anti-VEGF therapy, followed by a TAE protocol in which the interval for the next treatment is extended (by 2 weeks) for patients who demonstrate an absence of activity (8). Patients who remain quiescent 12 weeks from their prior treatment enter a “treatment pause” and are switched to PRN treatment; treatment is resumed based on a decline in vision or worsening on clinical exam and/or imaging studies. Using this approach, we reported that patients with nvAMD at the end of 1 year can be divided into 3 groups: (a) patients who require monthly treatment with anti-VEGF therapy (i.e., patients who fail treatment extension; ~20% of patients); (b) patients who require less-frequent but ongoing treatment with anti-VEGF therapy (~50% of patients); and (c) patients who can be successfully weaned off treatment with anti-VEGF therapy (~30% of patients) (8). Interestingly, neither the pre- nor posttreatment aqueous fluid levels of VEGF correlated with the need for more-frequent — or less-frequent — treatment with anti-VEGF therapy (8), suggesting that other vasoactive mediators may contribute to the response of patients with nvAMD to treatment.

Collectively, these observations emphasize the importance of ongoing efforts to identify other factors that contribute to the development of CNV and that may, therefore, serve as molecular biomarkers and/or influence the response of patients with nvAMD to anti-VEGF therapies. Of interest, the transcriptional activator hypoxia-inducible factor 1 (HIF-1) has been hypothesized to play a critical role in regulating the pathologic expression of numerous angiogenic mediators (including VEGF) that, together, promote ocular neovascularization (9, 10). In nvAMD, it is hypothesized that outer retinal ischemia due to interruption of oxygen delivery from the choriocapillaris to the overlying retinal pigment epithelium (RPE) results in HIF-1α accumulation (11). Indeed, it has been reported that the choriocapillaris underlying the macula becomes attenuated (and its function compromised) with aging (12). In patients with AMD, material (i.e., drusen) accumulates underneath and within the thickened RPE basement (Bruch’s) membrane, forming a physical barrier for O_2_ diffusion and further exacerbating the reduced oxygen delivery to the overlying RPE (13). This, in turn, is thought to result in the pathological accumulation of HIF-1α in the RPE of patients with nvAMD.

There is considerable preclinical evidence that HIF-1 participates in the regulation of VEGF expression and the development of CNV in patients with nvAMD, also known as wet AMD (14). Increased expression of HIF-1α has been reported in CNV membranes in AMD eyes (15, 16). Inhibition of HIF-1 in the RPE, using either pharmacologic (17) or RNA interference (RNAi) (18) strategies, decreases the size of CNV lesions in mouse models. We, therefore, set out to examine the contribution of HIF-regulated angiogenic factors, in addition to VEGF, in the promotion of CNV.

## Results

### Increased ANGPTL4 expression in patients with nvAMD who respond inadequately to anti-VEGF therapy.

**Figure 1:**
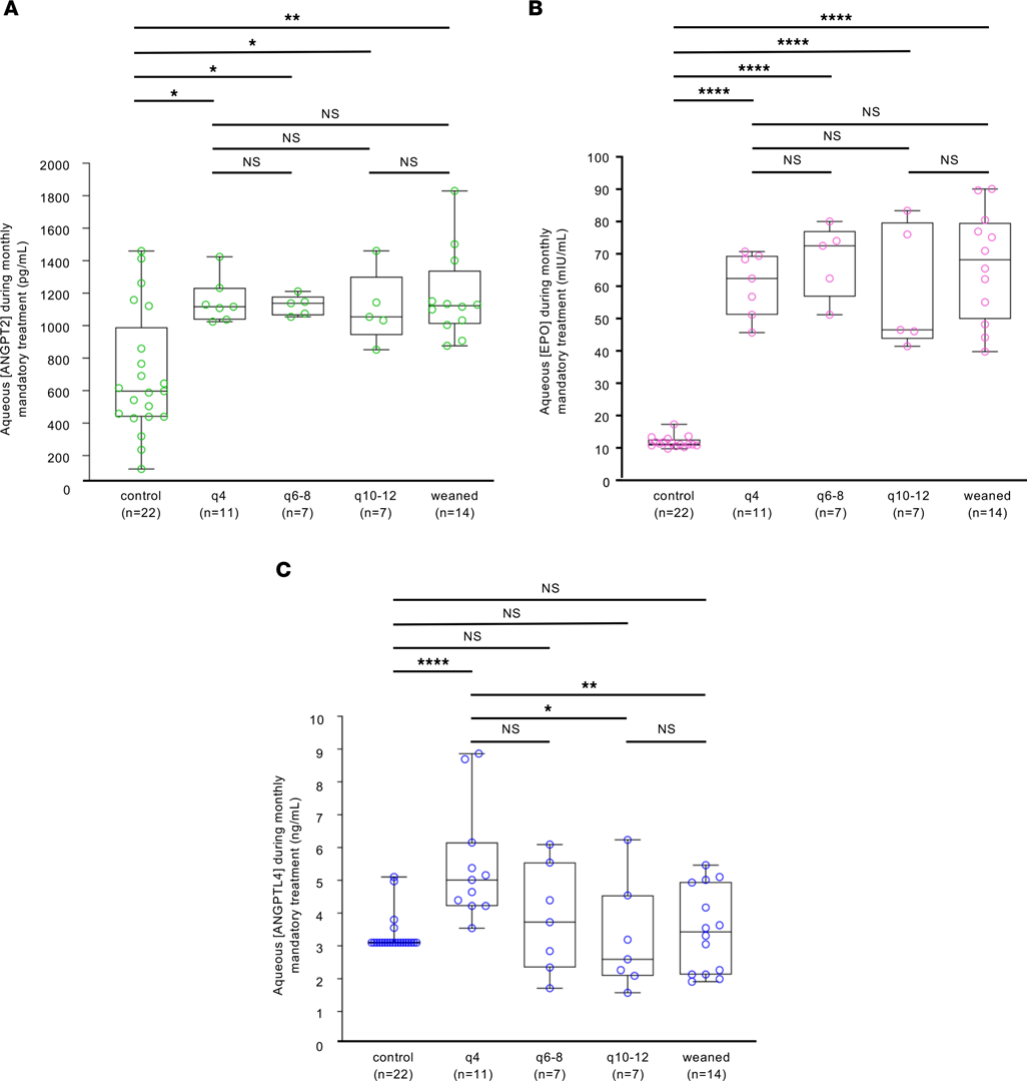
Aqueous fluid levels of HIF-regulated vasoactive mediators, ANGPT2, EPO, and ANGPTL4 in patients with nvAMD treated with anti-VEGF therapy. (**A**–**C**) Aqueous ANGPT2 (**A**), EPO (**B**), and ANGPTL4 (**C**) levels of patients during initial 3 monthly mandatory treatment phase of a treat-and-extend protocol for patients with increasing intervals between treatments at 12 months (from subset of TEP/M patients divided into groups based on the required treatment interval at the end of year 1 using the TEP/M protocol to wean patients from treatment; ref. 8). Patients extended to 12 weeks had treatment paused, and patients were considered “weaned” off treatment if treatment pause reached 30 weeks. Statistical analysis was performed using Wilcoxon rank sum test. control, non-AMD eyes; q4, patients requiring treatment every 4 weeks; q6–8, patients requiring treatment every 6–8 weeks; q10-12, patients requiring treatment every 10-12 weeks. *P < 0.05; **P < 0.01; ****P < 0.0001.

To identify vasoactive factors that could influence the response of these patients with nvAMD to anti-VEGF therapy, we shifted our attention upstream from VEGF to the transcription factor that regulates its expression, HIF-1, the master regulator of genes encoding angiogenic mediators (19). Evidence has converged on 1 HIF-regulated vasoactive mediator, angiopoietin 2 (ANGPT2), in ocular neovascular disease, and specifically in the development of CNV in patients with nvAMD (20). ANGPT2 acts synergistically with VEGF to promote angiogenesis but is antiangiogenic in the absence of VEGF (21). It has previously been reported that ANGPT2 promotes the development of vascular permeability and pathological angiogenesis in animal models of nvAMD (22, 23). Expression of ANGPT2 mRNA (24) and protein (25) has been detected in surgically excised CNV membranes from patients with nvAMD. This led to speculation that ANGPT2 expression may contribute to the modest response of some patients with nvAMD to anti-VEGF therapy (26, 27). Consequently, there are ongoing and recently completed clinical trials examining the safety and efficacy of therapies directly or indirectly targeting ANGPT2 and its downstream receptor, TIE2, in patients with nvAMD (28, 29). To determine if ANGPT2 expression influenced the response of patients with nvAMD to anti-VEGF therapy, we examined levels of ANGPT2 in patients treated with anti-VEGF therapy using the TEP/M protocol. We did not observe a difference in the aqueous fluid levels of ANGPT2 in patients who required monthly treatment with anti-VEGF therapy compared with patients who required less-frequent treatment or those who could be weaned off treatment (Figure 1A and Supplemental Table 1; supplemental material available online with this article; https://doi.org/10.1172/jci.insight.157896DS1). In patients who responded inadequately to anti-VEGF therapy, there were also no observed differences in the aqueous fluid levels of erythropoietin (EPO) (30, 31), another HIF-regulated vasoactive mediator that has also been previously implicated in the pathogenesis of nvAMD (Figure 1B). While these data do not rule out a role for therapies targeting ANGPT2 or EPO for the treatment of CNV, they suggest that additional vasoactive mediators may contribute to the inadequate response of some patients with nvAMD to current anti-VEGF therapies.

Another HIF-regulated angiogenic mediator, ANGPT-like 4 (ANGPTL4), has been implicated in pathological angiogenesis in ischemic retinal disease (32–34). We observed an increase in the levels of ANGPTL4 in the aqueous fluid of patients who required injections every 4 weeks (i.e., patients who failed treatment extension) using the TEP/M protocol compared with non-AMD controls (Figure 1C). However, increased aqueous ANGPTL4 levels were not detected in patients whose treatment interval could be extended to 6–8 weeks or 10–12 weeks, or who entered a treatment pause by the end of 1 year (Figure 1C). Moreover, the aqueous fluid levels of ANGPTL4 were lower in patients who could be extended to every 10–12 weeks or patients who entered a treatment pause by the end of 1 year compared with patients whose treatment interval could be extended (Figure 1C). Collectively, these data suggest that aqueous fluid levels of ANGPTL4 may help predict the response of patients with nvAMD to anti-VEGF therapy.

### ANGPTL4 is a biomarker for the response of patients with nvAMD to anti-VEGF therapy.

**Figure 2:**
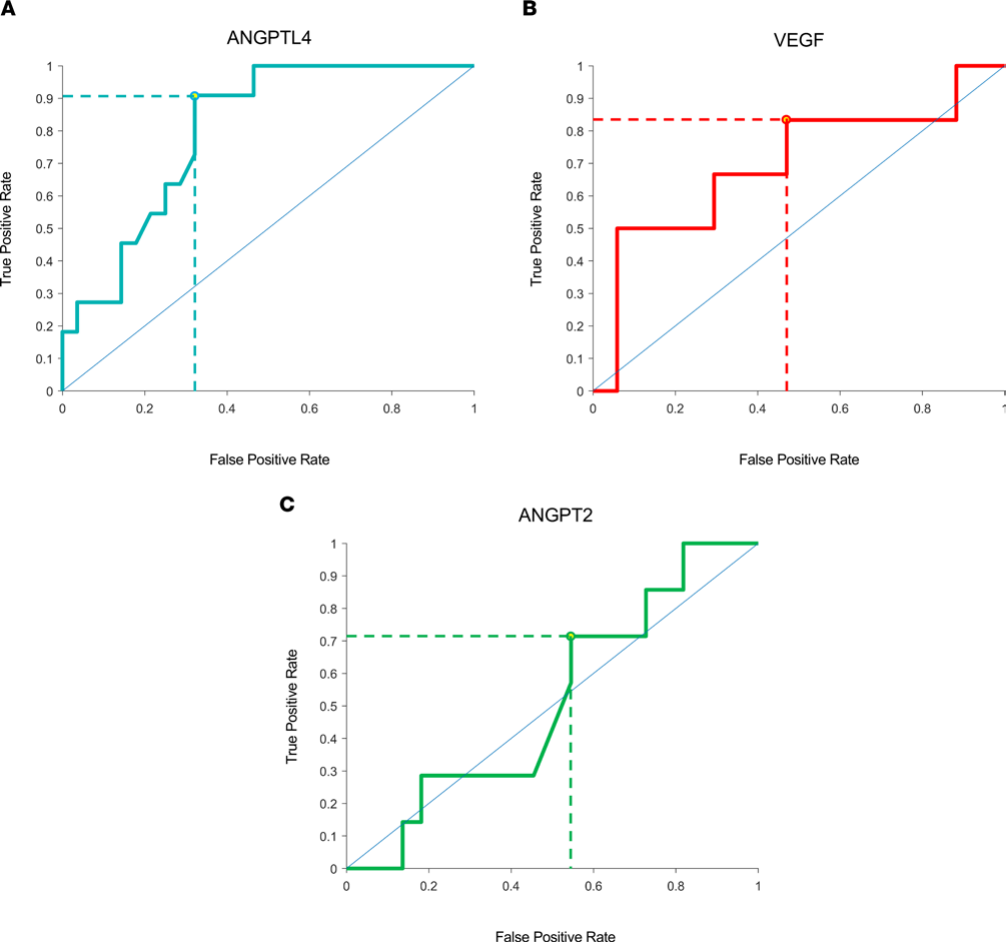
ROC curves for aqueous fluid levels of HIF-regulated vasoactive mediators, ANGPTL4, VEGF, and ANGPT2, in patients who require monthly treatment with anti-VEGF therapy. (**A**–**C**) ROC curves for HIF-regulated vasoactive mediators in TEP/M patients when predicting patients who required monthly treatment with anti-VEGF for ANGPTL4 (**A**), VEGF (**B**), and ANGPT2 (**C**). Optimal predictive values are denoted by dashed lines based on selected cutoff concentrations for each mediator: VEGF, 260 pg/mL; ANGPT2, 1100 pg/mL; and ANGPTL4, 4.22 ng/mL.

We next generated a receiving operating characteristics (ROC) curve to determine whether aqueous fluid levels of ANGPTL4 could be an effective biomarker for patients who require monthly treatment with anti-VEGF therapy. To this end, an ROC for aqueous ANGPTL4 was generated, and at a cut-off of 4.22 ng/mL, the sensitivity for predicting which patients would require monthly injection was 91% with a specificity of 68% (Figure 2A). Although the sample size was small, this suggests that ANGPTL4 may be an effective biomarker for predicting the response of patients with nvAMD to treatment with anti-VEGF therapy. ROC curves for VEGF (with a cut-off of 260 pg/mL) and ANGPT2 (with a cut-off of 1.10 ng/mL) had lower sensitivity (83% and 71 %, respectively) and specificity (53% and 45%, respectively) compared with ANGPTL4 (Figure 2, B and C). However, by combining the results for VEGF and ANGPTL4, the sensitivity for predicting which patients would require monthly injection was 76% with a specificity of 85% (Supplemental Table 2). These results suggest that pretreatment levels of VEGF and early levels of ANGPTL4 could help predict how patients with nvAMD will respond to treatment with anti-VEGF therapy.

### Increased ANGPTL4 expression in the aqueous fluid of patients with nvAMD with active CNV.

**Figure 3:**
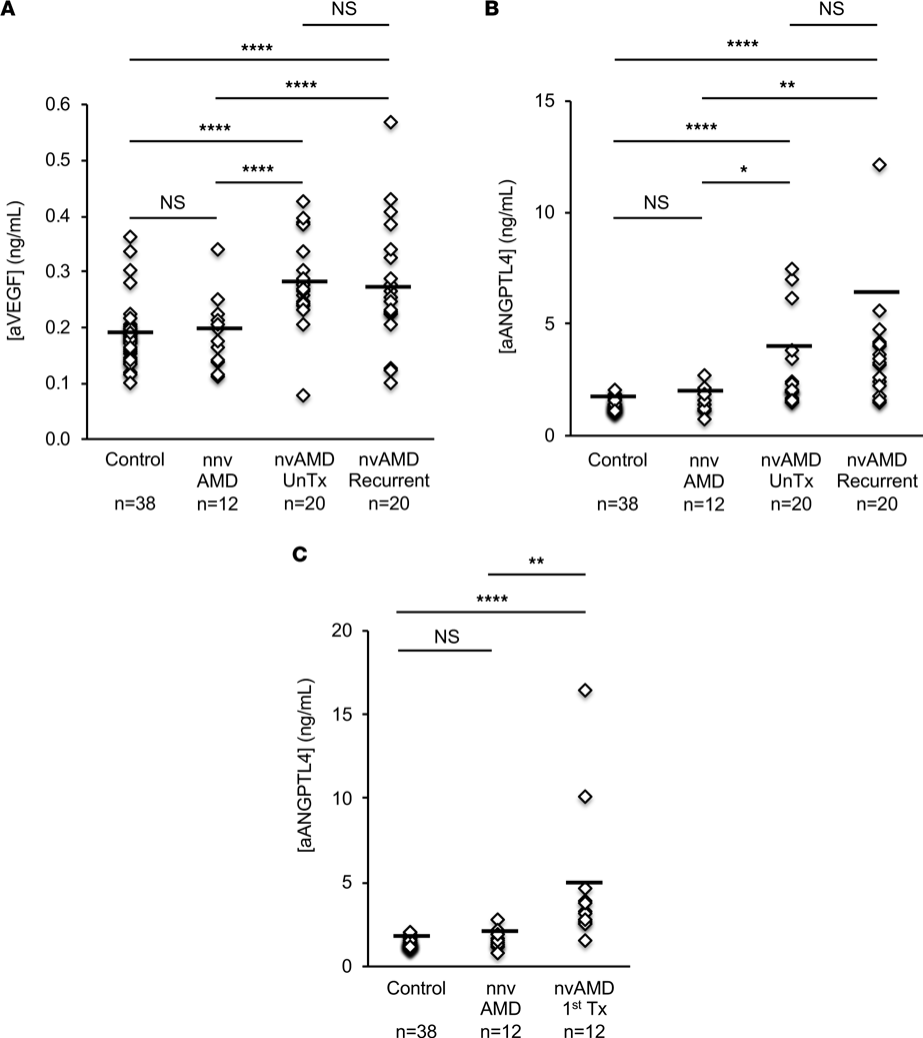
Expression of VEGF and ANGPTL4 in aqueous fluid from patients with nvAMD treated in the clinic for active CNV. (**A** and **B**) Aqueous fluid levels of VEGF (**A**) and ANGPTL4 (**B**) in treatment-naive patients with active nvAMD (i.e., patients with nvAMD who have never received anti-VEGF therapy; nvAMD UnTx) and patients with nvAMD previously treated with anti-VEGF therapy 12 or more weeks prior to sample collection (nvAMD Recurrent) compared with patients with nnvAMD and non-AMD (Control) patients. Aqueous fluid samples with ANGPTL4 > 15 ng/mL are not displayed to adequately demonstrate the variability within the nvAMD samples; see Supplemental Figure 1, A and B, for all samples tested. (**C**) Aqueous fluid levels of ANGPTL4 in patients with nvAMD treated with their first anti-VEGF therapy within 4–6 weeks of sample collection “nvAMD 1st Tx” compared with patients with nnvAMD and non-AMD (Control) patients. Kruskal-Wallis with Dunn’s multiple-comparison test. *P < 0.05; **P < 0.01; ****P < 0.0001.

To further characterize the expression of ANGPTL4 in patients with nvAMD being treated in the clinic for active CNV, we obtained aqueous fluid samples from untreated, newly diagnosed patients with nvAMD (nvAMD UnTx) who were initiating treatment with anti-VEGF therapy and examined the levels of ANGPTL4 compared with aqueous fluid samples obtained prior to the initiation of cataract surgery from patients without AMD (Control) and patients with nonneovascular AMD (nnvAMD), or “dry” AMD (Supplemental Table 3). The mean levels of VEGF and ANGPTL4 were not significantly different between control patients without AMD and patients with nnvAMD (Figure 3, A and B). However, we observed significantly increased expression of both VEGF (1.5-fold; *P* < 0.0001) and ANGPTL4 (2.7-fold; *P* < 0.0001) in the aqueous fluid of treatment-naive (i.e., no history of anti-VEGF therapy) nvAMD eyes compared with control eyes, as well as compared with nnvAMD eyes (1.5-fold and 2.4-fold, respectively; both *P* < 0.01).

We next examined whether ANGPTL4 levels were also increased in aqueous fluid from patients who had a history of anti-VEGF therapy but had not been treated in ≥ 12 weeks prior to sample collection and those who had evidence of active CNV (nvAMD Recurrent; Supplemental Table 3). We observed increased mean VEGF (1.5-fold; *P* < 0.0001) and ANGPTL4 (4.6-fold; *P* < 0.0001) levels in the aqueous fluid of nvAMD Recurrent patients compared with control eyes, as well as compared with eyes of patients with nnvAMD (1.4-fold and 4.1-fold, respectively; *P* < 0.0001 and *P* < 0.01, respectively) (Figure 3, A and B, and Supplemental Figure 1). Collectively, these results suggest that ANGPTL4 may be a therapeutic target for patients with newly diagnosed and recurrent CNV.

For therapies targeting ANGPTL4 to be effective in patients already receiving currently available therapies targeting VEGF, ANGPTL4 levels would need to remain increased despite receiving anti-VEGF treatments. To assess the levels of ANGPTL4 after initiation of intravitreal anti-VEGF therapy, aqueous fluid samples from patients were obtained 4–6 weeks after their first treatment with anti-VEGF therapy and were analyzed (nvAMD first Tx; Supplemental Table 3). Mean ANGPTL4 levels in this group remained increased compared with control and nnvAMD eyes (3.4-fold and 3-fold, respectively; *P* < 0.0001 and *P* < 0.01, respectively; Figure 3C). Collectively, these studies indicate that ANGPTL4 expression is increased in patients with nvAMD with active CNV, including patients who have either a recent or remote history of anti-VEGF therapy.

### ANGPTL4 expression in CNV lesions in nvAMD eyes.

**Figure 4:**
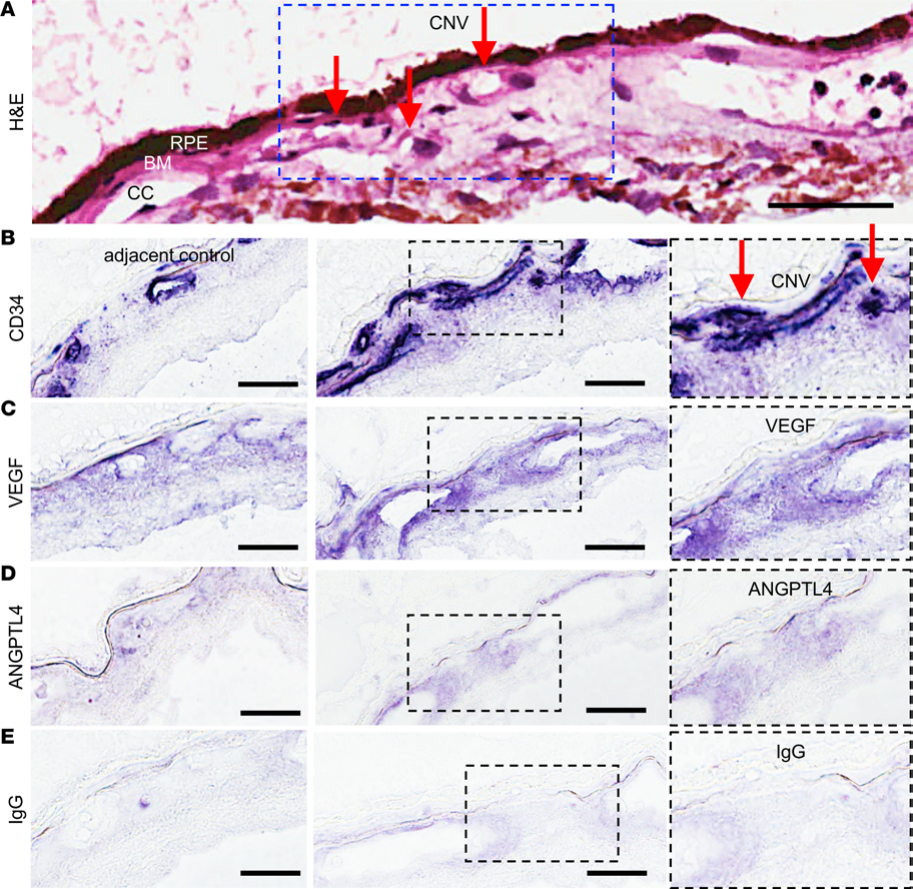
Expression of VEGF and ANGPTL4 in autopsy eyes from patients with known nvAMD. (**A**) Representative images from H&E staining of a CNV lesion in an autopsy eye from a patient with known nvAMD. Red arrows point to CNV vessels that have broken through (and are anterior to) Bruch’s membrane (BM). (**B**) Representative images from immunohistochemical analysis for CD31 within the area of the CNV membrane (CNV; right) or adjacent tissue without active CNV (adjacent control; left). (**C** and **D**) Representative images from immunohistochemical analysis for VEGF and ANGPTL4 within the area of the CNV membrane or in adjacent control. Inset demonstrates magnified view of staining within CNV lesion. (**E**) IgG is used as a negative control. RPE, retinal pigment epithelium; BM, Bruch’s membrane; CC, choriocapillaris. Scale bars: 25 μm.

To confirm that ANGPTL4 expression was increased within CNV lesions in patients with nvAMD, we examined autopsy eyes from patients with a known diagnosis of nvAMD with active CNV by histopathology (Figure 4, A and B); adjacent tissue without evidence of CNV was used as a control. Expression of VEGF by the RPE has been reported to be important for maintaining the health of the choriocapillaris (12, 35, 36). Accordingly, IHC analysis of CNV lesions demonstrated light staining for VEGF in the RPE and choroid without active CNV (Figure 4C). Strong staining for VEGF was detected within CNV lesions (Figure 4C), as has previously been described (36). We observed a similar pattern for ANGPTL4 expression, with light staining within the choroid without active CNV but strong staining for ANGPTL4 within CNV lesions in 4 of 4 nvAMD eyes examined (Figure 4D). IgG was used as a negative control (Figure 4E). Collectively, these data demonstrate that expression of ANGPTL4, similar to VEGF, is observed in the choriocapillaris and within CNV lesions in the eyes of patients with nvAMD.

### HIF-1–dependent ANGPTL4 expression in the laser-induced CNV model of nvAMD.

**Figure 5:**
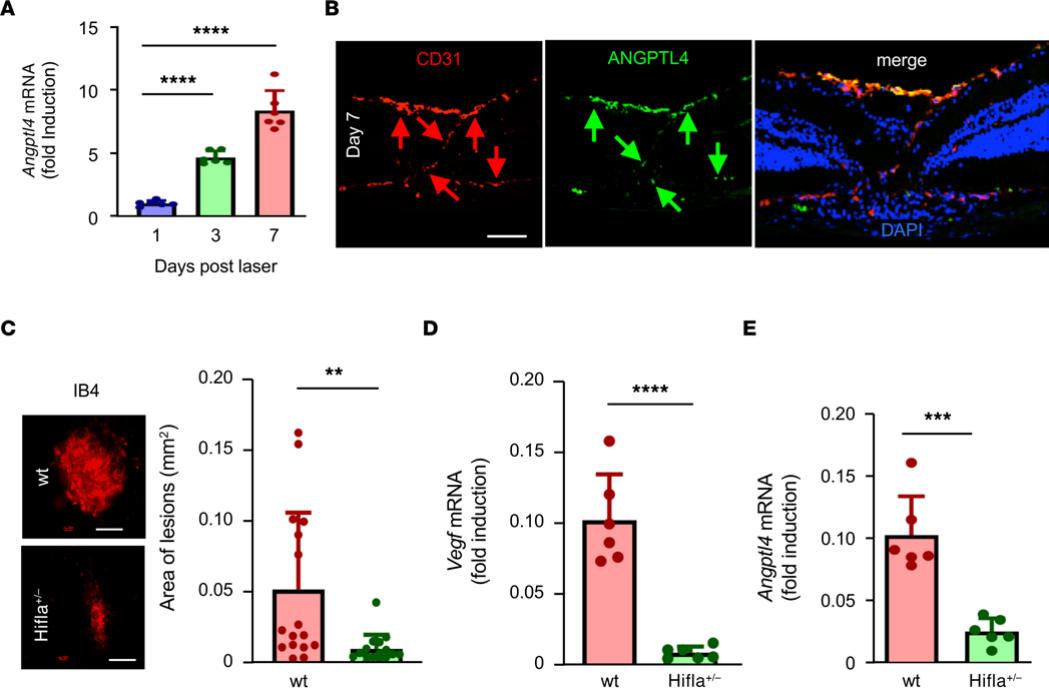
HIF-dependent expression of ANGPTL4 in laser-induced CNV lesions in mice. (**A**) Expression of *Angptl4* mRNA (qPCR) in RPE/choroidal lysates from laser CNV eyes over time. (**B**) Expression of ANGPTL4 protein (green arrows) in laser CNV lesions (labeled with CD31; red arrows) 7 days following laser treatment. (**C**) Size of CNV lesions in *Hif1a*^+/–^ mice compared with WT littermate controls. (**D** and **E**) Expression of *Vegf* (**D**) and *Angptl4* (**E**) mRNA (qPCR) in RPE/choroidal lysates from laser CNV eyes in *Hif1a*^+/–^ mice compared with WT littermate controls. *n* = 3–6 animals. Blue nuclear staining with DAPI. Scale bars: 60 μm (**B**) or 200 μm (**C**). Student’s *t* test. **P < 0.01; ***P < 0.001; ****P < 0.0001.

To examine further whether ANGPTL4 directly contributes to the development of CNV in patients with nvAMD, we employed a mouse model of CNV, in which a laser is used to rupture Bruch’s membrane (37). The laser CNV model has proved to be a powerful tool to examine molecular events underlying the development of CNV (38). We observed increased expression of *Angptl4* mRNA over time in the RPE/choroid of lasered eyes compared with nonlasered contralateral (Control) eyes (Figure 5A). Examination of laser CNV lesions by immunofluorescence demonstrated ANGPTL4 expression within CNV lesions, extending from the RPE to the overlying CNV tissue (Figure 5B), similar to what was observed in CNV lesions from patients with nvAMD (Figure 4D).

We next set out to determine whether HIF-1α expression influenced ANGPTL4 expression in CNV lesions. To this end, we used mice that were heterozygous for a KO allele at the *Hif1a* locus (*Hif1a*^+/–^) (39). Basal levels of HIF-1α are relatively normal in *Hif1a*^+/–^ mice, whereas in response to ischemia, HIF-1α expression is largely unchanged in *Hif1a*^+/–^ mice but potently stimulated in WT littermate controls (40). CNV lesions in *Hif1a*^+/–^ mice were small compared with WT littermate controls (Figure 5C). This corresponded to a marked decrease in *Vegf* and *Angptl4* mRNA expression (Figure 5, D and E).

### Synergism between VEGF and ANGPTL4 in the pathogenesis of CNV.

**Figure 6:**
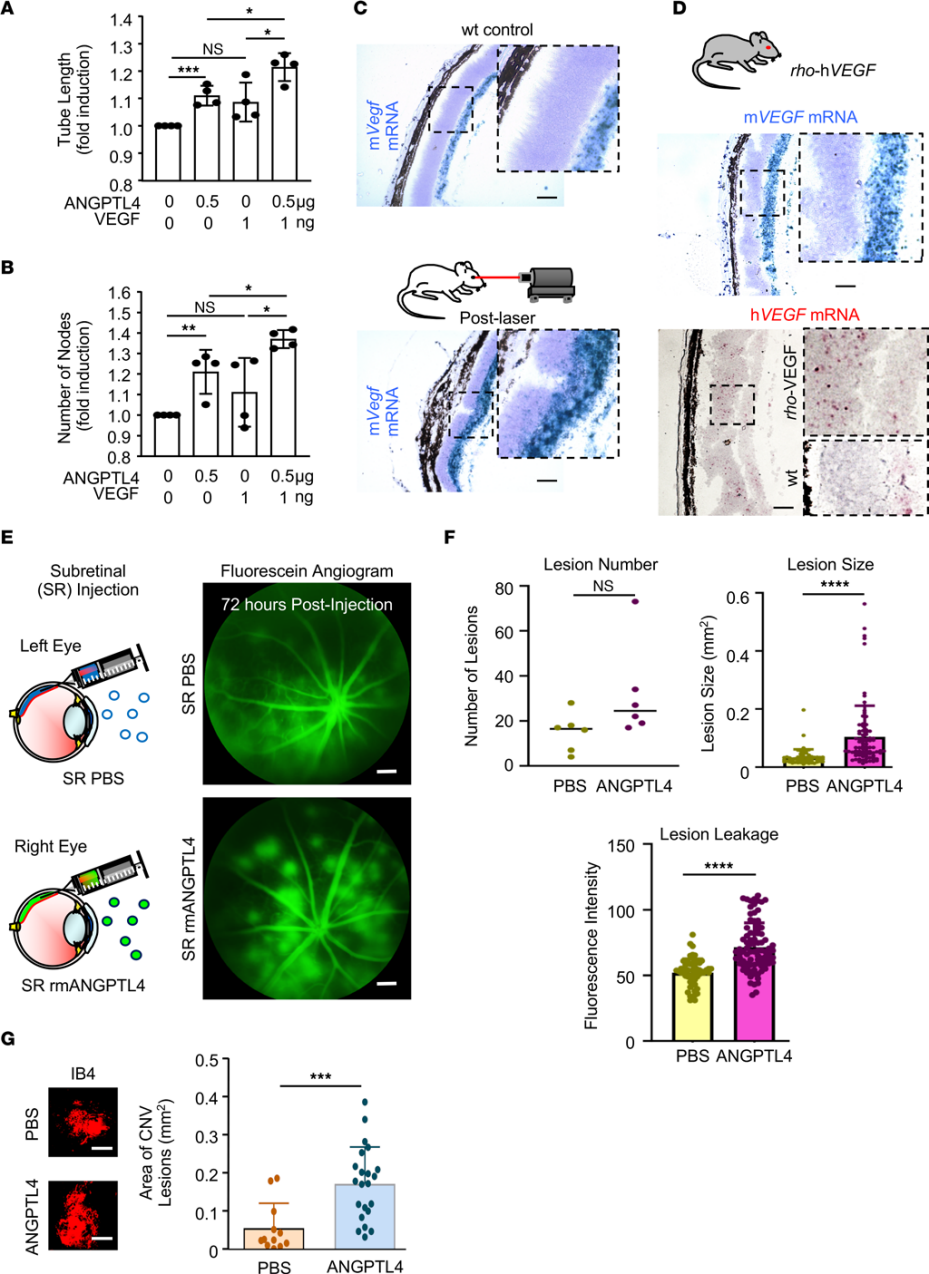
ANGPTL4 enhances the ability of VEGF to promote CNV lesions in mice. (**A** and **B**) Endothelial cell tubule length (**A**) and number of nodes (**B**) in response to treatment with rhANGPTL4, rhVEGF, or both. (**C**) Expression of murine *Vegf* (m*Vegf*) mRNA (in situ hybridization) in control (WT) animals (above) and 7 days following treatment with laser (below). (**D**) Expression of m*Vegf* (blue) and human *VEGF* (h*VEGF*; red) mRNA in *rho*-h*VEGF* transgenic mice. (**E**) Schematic (left) demonstrating subretinal injection with PBS (above) or rmANGPTL4 (below) in *rho*-h*VEGF* mice. Fluorescein angiogram (FA) demonstrating fluorescein leakage from CNV lesions 72 hours following subretinal injection of PBS (above) or rmANGPTL4 (below). (**F**) The number and size of, and leakage from CNV lesions on FA. (**G**) Size of CNV lesions on choroidal flat mounts stained with isolectin 72 hours after following subretinal injection of PBS or rmANGPTL4. *n* = 6–9 animals. Scale bars: 100 μm (**C** and **D** [above]) and 200 μm (**D** [below], **E**, and **G**). Wilcoxon rank sum test and Student’s *t* test. *P < 0.05; **P < 0.01; ***P < 0.001; ****P < 0.0001.

It has previously been reported that ANGPTL4, along with VEGF, stimulates vascular permeability (34, 41) and pathological angiogenesis (32, 42) in preclinical models of ischemic retinal disease. We hypothesized that CNV lesions may similarly be dependent on both VEGF and ANGPTL4 expression. To test this hypothesis, we first analyzed the effect of ANGPTL4 on tubule formation (an in vitro proxy for angiogenesis) in an immortalized mouse retinal endothelial cell (iREC) line (43) in the absence or presence of VEGF. Recombinant murine ANGPTL4 (rmANGPTL4) induced iREC tubule formation (increasing tube length and number of nodes; Figure 6, A and B), similar to rmVEGF. Interestingly, treatment of iRECs with both factors resulted in a greater effect than either factor alone (Figure 6, A and B).

To examine in vivo the contribution of ANGPTL4 to the development of CNV, we took advantage of a transgenic model of CNV. In the laser CNV model, increased *Vegf* mRNA expression was observed in the region of laser injury (Figure 6C). The increased expression of *Vegf* mRNA in the outer retina of mice was reproduced in the *rho-*h*VEGF* transgenic mouse (Figure 6D), in which the human VEGF is constitutively expressed under the rod-specific rhodopsin promoter (44). The *rho-*h*VEGF* transgenic mouse has previously been reported to reproduce CNV-like lesions that are observed as early as 3 weeks of age (44). To investigate the ability of ANGPTL4 to modulate VEGF-induced CNV, we administered subretinal injections of rmANGPTL4 in 2-week-old *rho*-h*VEGF* mice (Figure 6E). Although the modest increase in CNV lesion number 72 hours after subretinal injection of rmANGPTL4 compared with PBS control was not significant, we observed a marked increase in CNV lesions size and leakage, as assessed by fluorescein angiography (FA) (Figure 6F). The increase in CNV lesions size after subretinal injection of rmANGPTL4 was corroborated using fluorescein-conjugated isolectin B4 to stain CNV lesions on choroidal flat mounts 72 hours following injection, compared with PBS control (Figure 6G). Collectively, these results suggest that ANGPTL4 enhances the size of — and leakage from — CNV lesions induced by forced expression of VEGF in mice.

### Targeting ANGPTL4, VEGF, or both for treating CNV in mice.

**Figure 7:**
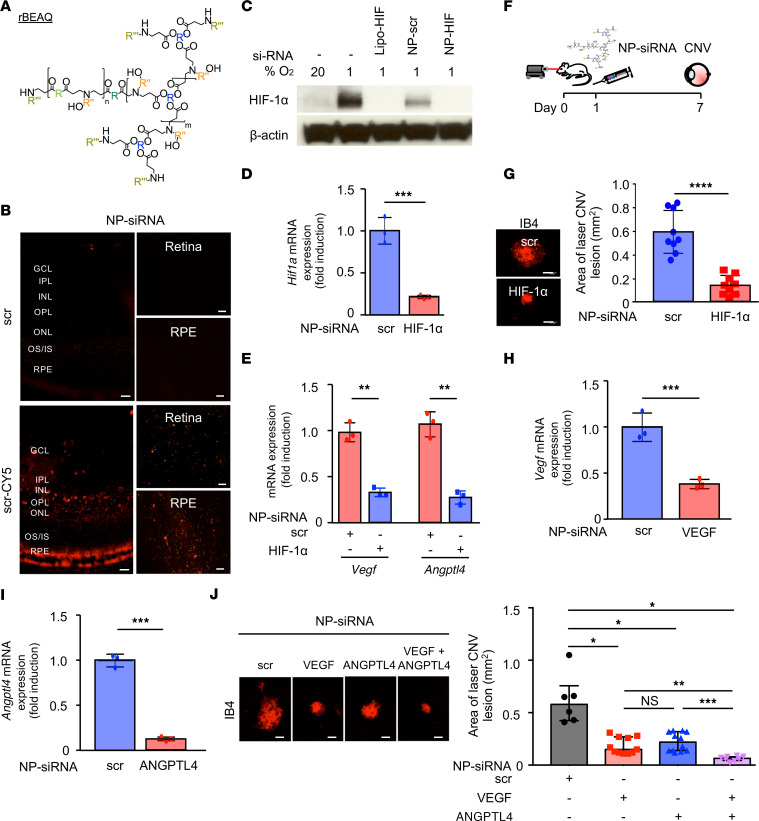
In vivo nanoparticle-mediated siRNA targeting ANGPTL4 and VEGF is more effective than targeting either angiogenic factor alone for the treatment of laser CNV mice. (**A**) rBEAQ polymer for in vivo delivery of siRNA. (**B**) Expression of fluorophore in cross section (left) and neurosensory retina (Retina) or RPE/choroidal (RPE) flat mounts (right) of mice 1 day following intravitreal injection with NP-scr or NP-scr conjugated to Cy5. (**C**) Expression of HIF-1α protein (WB) in lysates from primary mouse RPE cells treated with 1% O_2_ in the presence of nanoparticle-encapsulated siRNA targeting *Hif1a* (NP-HIF) versus scrambled control (NP-scr) for 72 hours. Standard transfection with Lipofectamine RNAiMAX-encapsulated siRNA targeting *Hif1a* (Lipo-HIF) was used as a positive control. (**D** and **E**) *Hif1a* (**D**), *Vegf*, and *Angptl4* (**E**) mRNA expression (qPCR) in RPE/choroidal lysates 5 days following a single intravitreal injection with siRNA targeting *Hif1a* (NP-HIF) versus NP-scr in mice. (**F**) Schematic of laser CNV model in which a single dose of NP-HIF or NP-scr control is administered by intravitreal injection 1 day after laser treatment; eyes were enucleated for analysis on day 7. (**G**) Size of CNV lesion in mice treated with NP-scr or NP-HIF. (**H** and **I**) *Vegf* (**H**) and *Angptl4* (**I**) mRNA expression (qPCR) in RPE/choroidal lysates 5 days following a single intravitreal injection with siRNA targeting *Vegf* (NP-VEGF) or *Angptl4* (NP-ANGPTL4), respectively. (**J**) Size of CNV lesion in mice treated with NP-scr, NP-VEGF, NP-ANGPTL4, or both NP-VEGF and NP-ANGPTL4 administered by intravitreal injection 1 day after laser treatment; eyes were enucleated for analysis on day 7. *n* = 3–6 animals. GCL, ganglion cell layer; IPL, inner plexiform layer; INL, inner nuclear layer; OPL, outer plexiform layer; ONL, outer nuclear layer; IS/OS, inner/outer segments; RPE, retinal pigment epithelium. Scale bars:25 μm (**B**) and 100 μm (**G** and **J**). Student’s *t* test (**D**, **E**, and **G**–**I**) and Kruskal-Wallis with Dunnett’s T3 correction (**J**). *P < 0.05; **P < 0.01; ***P < 0.001; ****P < 0.0001.

To examine whether ANGPTL4 may be required for the development of CNV, we took advantage of a potentially novel RNAi approach to knock down expression of genes in the mouse eye (45). We generated siRNA-encapsulating nanoparticles using reducible branched ester amine quadpolymers (rBEAQs; Figure 7A and Supplemental Figure 2) (46). These biodegradable nanoparticles are designed to escape the endosomal compartment and then release siRNA cargo in an environmentally triggered manner upon cleavage of disulfide bonds in the polymer backbone in the reducing cytosolic environment. Intraocular injection with the nanoparticle complexed with scrambled siRNA (NP-scr) conjugated to a fluorophore (Cy5) demonstrated effective transfection of retinal cells in the outer retina, as well as the underlying RPE, 24 hours after a single intravitreal injection (Figure 7B).

We used these biodegradable polymeric nanoparticles to deliver HIF-1 siRNA (NP-HIF) and observed robust knockdown of HIF-1α protein in hypoxic primary mouse RPE cells (Figure 7C), similar to transfection utilizing a lipid-based commercial reagent encapsulating HIF-1α siRNA (Lipo-HIF). Accordingly, we observed potent knockdown of *Hif1a* mRNA expression in vivo at 5 days after intraocular administration of NP-HIF (Figure 7D), resulting in a reduction in *Vegf* and *Angptl4* mRNA expression compared with scrambled siRNA (NP-scr) control (Figure 7E). To assess the effect of NP-HIF on CNV lesions, we treated mice with a single intraocular injection of NP-HIF or NP-scr at day 1 (Figure 7F). NP-HIF potently inhibited the development of CNV in response to laser injury (Figure 7G), similar to what was observed within *Hif1a*^+/–^ mice (Figure 5C).

To evaluate the contribution of ANGPTL4 to the development of CNV in mice, we next encapsulated *Angptl4* siRNA (NP-ANPGLT4); *Vegf* siRNA (NP-VEGF) was used as a control. Intravitreal injection with NP-VEGF or NP-ANGPTL4 resulted in knockdown of *Vegf* (Figure 7H) or *Angptl4* (Figure 7I) mRNA expression, respectively. Treatment of laser CNV mice with a single intraocular injection of NP-ANGPTL4 1 day after laser injury resulted in decreased area of CNV lesions on day 7 compared with NP-scr control (Figure 7J). There was no difference in CNV lesion area in animals treated with NP-VEGF compared with NP-ANGPTL4 (Figure 7J), indicating that therapies targeting ANGPTL4 are as effective as therapies targeting VEGF in this model. To evaluate the therapeutic potential of combining anti-VEGF and anti-ANGPTL4 therapies, we performed intravitreal injections of both NP-VEGF and NP-ANGPTL4 together. This resulted in a further decrease in CNV lesion area compared with injection with either NP-VEGF or NP-ANGPLT4 alone (Figure 7J). Collectively, these results suggest that therapies targeting ANGPTL4 could be effective alone but would also enhance the efficacy of current anti-VEGF therapies for the treatment of patients with nvAMD.

### Targeting both VEGF and ANGPTL4 with a soluble receptor, sNRP1, effectively inhibits CNV in mice.

**Figure 8:**
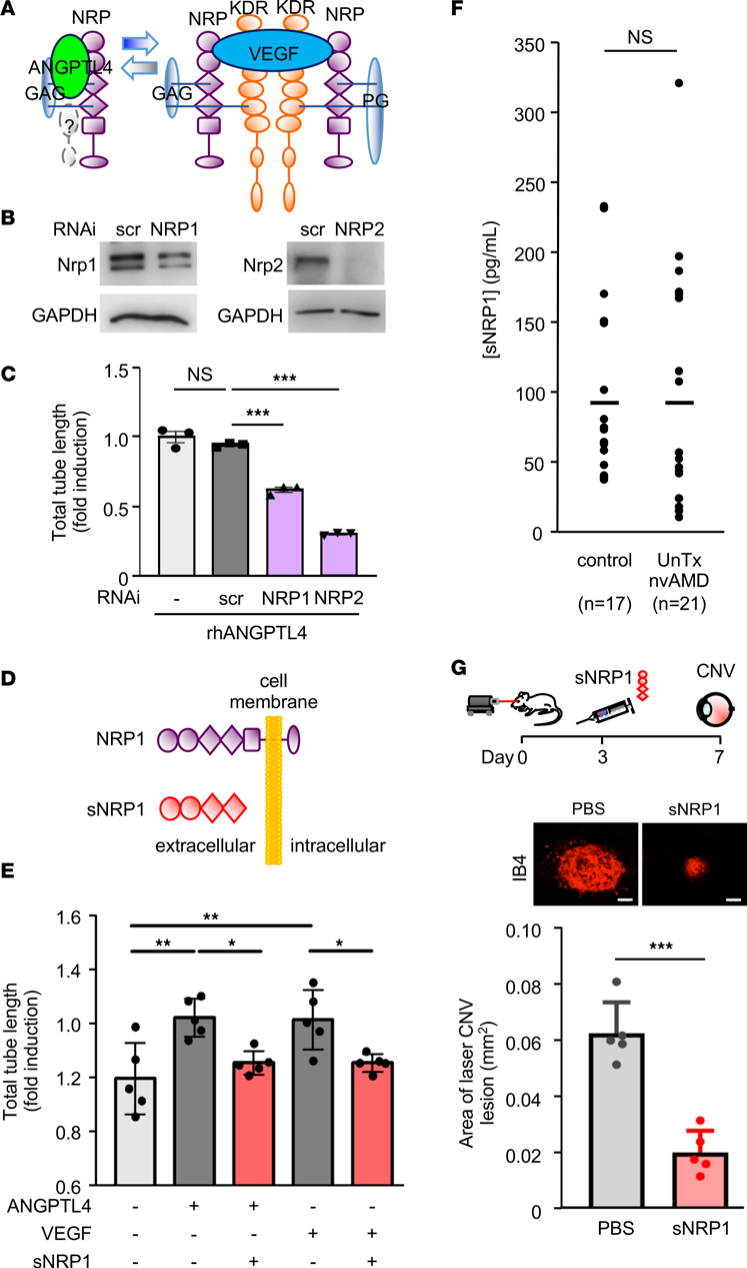
sNRP1 reduces the promotion of angiogenesis by ANGPTL4 and inhibits CNV in mice. (**A**) Schematic demonstrating binding of ANGPTL4 and VEGF to the endothelial cell receptors, neuropilin 1 (NRP1) and NRP2. (**B** and **C**) Knockdown of NRP1 or NRP2 (**B**) inhibits the promotion of human umbilical vein endothelial cell (HUVEC) tubule formation by rhANGPTL4 (**C**). (**D**) Schematic comparing NRP1 and sNRP1; the latter lacks the transmembrane domain and is, therefore, soluble. (**E**) sNRP1 inhibits the promotion of iREC tubule formation by rhANGPTL4 and rhVEGF. (**F**) Expression of sNRP1 in the aqueous fluid of patients with treatment-naive (UnTx) nvAMD compared with non-AMD controls. (**G**) Schematic of laser CNV model in which a single dose of rhsNRP1 control is administered by intravitreal injection 3 days after laser treatment; eyes were enucleated for analysis on day 7. *S*ize of CNV lesion in mice treated with PBS or rhsNRP1. *n* = 3–6 animals. Scale bars: 100 μm. One-way ANOVA with Bonferroni correction (**C** and **E**) and Student’s *t* test (**F** and **G**). *P < 0.05; **P < 0.01; ***P < 0.001.

We recently demonstrated that the endothelial cell receptor, neuropilin (NRP), binds to ANGPTL4 to promote Rho activation and increase vascular permeability (41) (Figure 8A). We therefore knocked down NRP1 and NRP2 expression with RNAi in human umbilical vein endothelial cells (HUVECs) to examine the role of this receptor on the promotion of pathological angiogenesis mediated by ANGPTL4 (Figure 8B). We observed impaired rhANGPTL4-mediated endothelial cell tubule formation following knockdown of NRP1 or NRP2 (Figure 8C), indicating that NRPs are also required for pathological angiogenesis mediated by ANGPTL4.

Soluble NRPs (sNRPs) are naturally occurring fragments of NRPs that lack the transmembrane and intracellular domains (Figure 8D). They are expressed independently from intact NRPs and function as endogenous inhibitors of the biological effects of NRP signaling by acting as traps for the NRP ligands (47). sNRP1 has previously been reported to inhibit the binding of VEGF to full-length NRP1, and both sNRP1 and sNRP2 have been shown to inhibit tumor angiogenesis and growth (48, 49). We recently reported that sNRP1 also prevents binding of ANGPTL4 to NRP1 and NRP2 and inhibits vascular permeability mediated by ANGPTL4 (41). Treatment of iRECs with recombinant human sNRP1 resulted in a potent inhibition of ANGPTL4-induced iREC tubule formation, similar to what was observed for VEGF-mediated tubule formation (Figure 8E). These results suggested that sNRP could provide an effective approach to block the effects of both ANGPTL4 and VEGF.

**Figure 9:**
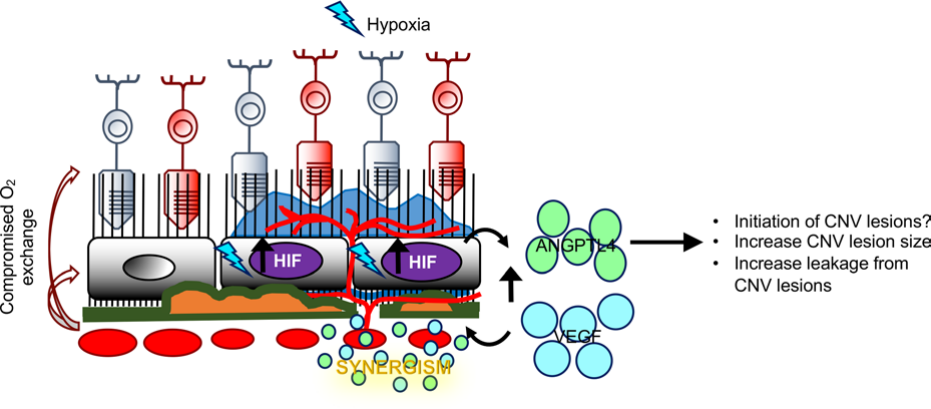
Schematic demonstrating the synergistic contribution of VEGF and ANGPTL4 in the development of CNV in patients with nvAMD. In the aging eyes of patients with AMD, collapse of the choriocapillaris combined with thickening of Bruch’s membrane and drusen deposits impedes delivery of oxygen to the overlying RPE. This, in turn, results in relative ischemia, the accumulation of HIF-1α, and increased expression of VEGF and ANGPTL4. VEGF and ANGPTL4 cooperate to promote the development of CNV.

Since sNRP1 is an endogenous inhibitor of ANGPTL4 and VEGF, we next examined whether its expression was influenced by the presence of CNV in the eyes of patients with nvAMD. While expression of sNRP1 was detected in the eyes of patients with nvAMD, it was not increased compared with control patients without AMD (Figure 8F). Collectively, these observations exposed a potential therapeutic approach for the inhibition of CNV in patients with nvAMD using exogenous sNRP1. We therefore examined the effect of treating CNV in mice with recombinant human sNRP1 (rhsNRP1). A single intraocular injection of rhsNRP1 was able to inhibit CNV compared with control mice that were treated with vehicle (PBS) (Figure 8G). Collectively, these studies demonstrate that VEGF and ANGPTL4 contribute synergistically to the promotion of CNV lesions in patients with nvAMD (Figure 9) and suggest that therapies targeting both ANGPTL4 and VEGF will be the most effective therapeutic approach for the treatment of nvAMD.

## Discussion

Despite affecting only 10% of patients with AMD, CNV is the cause of severe vision loss in 90% of AMD patients (50). Over the last 20 years, a large body of data has demonstrated that VEGF plays a fundamental role in the pathogenesis of CNV in patients with nvAMD (51). This finding led to the development of therapies that specifically target VEGF and ushered in a new era in the treatment of this vision-threatening disease (50). Anti-VEGF therapies have had a remarkable impact on patients with nvAMD who previously suffered major vision loss from edema, bleeding, or scarring caused by CNV (3). Nonetheless, less than half of patients with nvAMD treated with anti-VEGF therapies have a major improvement in visual acuity (i.e., a gain of at least 15 letters — or 3 lines — on the ETDRS visual acuity chart), despite monthly or bimonthly injections. Moreover, the majority of patients who do respond to anti-VEGF therapy still have intraretinal or subretinal fluid (50). These studies suggest that additional vasoactive factors contribute to CNV pathogenesis and vision loss in patients with nvAMD.

In this regard, studies using animal models have demonstrated that expression of a constitutively active form of the transcription factor HIF-1α was sufficient to promote ocular neovascularization in vivo (52), while expression of VEGF alone was not sufficient to mediate this effect (53–55). These results implicate additional HIF-regulated angiogenic factors in the promotion of pathological neovascularization in the eye. Here, we examine the contribution of HIF-regulated angiogenic factors, in addition to VEGF, in the promotion of CNV.

Using a hybrid of TAE and PRN protocols, which we designated TEP/M, we recently reported that ~30% of patients with nvAMD can be safely weaned off anti-VEGF therapy within 1 year (8). However, ~20% of patients with nvAMD failed treatment extension, requiring intraocular injections with anti-VEGF therapy every 4 weeks. Most of these patients still had fluid, despite this aggressive treatment regimen (8). These results demonstrate that a subset of patients with nvAMD are less sensitive to currently available anti-VEGF therapies and implicate other vasoactive mediators in the promotion of CNV in these patients. We demonstrate here that aqueous fluid levels of ANGPT2, a key target for emerging therapies for nvAMD (26–29, 56–58), were unchanged in patients who responded well — or poorly — to anti-VEGF therapy. Conversely, aqueous fluid levels of another HIF-regulated vasoactive factor, ANGPTL4, correlated inversely with patients’ response to anti-VEGF therapy. Levels of ANGPTL4 in the aqueous fluid of patients with nvAMD who required treatment every 4 weeks were higher than patients with nvAMD who required treatment every 10–12 weeks injections or those patients who entered a treatment pause. These data suggest that patients with higher aqueous fluid levels of ANGPTL4 may be better suited for longer-acting anti-VEGF agents. Conversely, patients with low levels of ANGPTL4 may be adequately treated with currently available anti-VEGF therapies. Additional studies will be needed to determine if aqueous fluid levels of ANGPTL4 (and VEGF) at the time of treatment initiation could be used as biomarkers to help predict which patients would benefit from long-acting anti-VEGF therapies.

In addition to its role as a potential biomarker, we further observed that ANGPTL4 contributes to the size of, and leakage from, CNV lesions induced by VEGF overexpression in mice, supporting a synergistic role for ANGPTL4 — in combination with VEGF — in the promotion of CNV (Figure 9). We have previously demonstrated that ANGPTL4 can promote both angiogenesis (32, 42) and vascular permeability (34, 41, 59), the latter through promotion of the Rho/ROCK pathway (41). In addition to regulating angiogiogenesis, ANGPTL4 has also been reported to regulate lipid metabolism (60) and inflammation (61), both of which have also been implicated in the pathogenesis of AMD (62, 63). Additional studies will be necessary to determine the mechanisms by which ANGPTL4 contributes to the development of CNV in patients with nvAMD.

Although we did not observe a correlation between the size of CNV lesions in AMD patients and the aqueous fluid levels of ANGPTL4, as has previously been reported (64), levels of ANGPTL4 remained increased in the aqueous fluid of patients with nvAMD being treated for active CNV compared with control patients without AMD, or patients with nnvAMD. Increased ANGPTL4 levels were observed in patients with a recent (within 4–6 weeks) or remote (>12 weeks) history of anti-VEGF therapy, suggesting that elevated ANGPTL4 levels were not dependent on elevated VEGF levels and that therapies targeting ANGPTL4 could be used in conjunction with current therapies targeting VEGF. Persistent expression of ANGPTL4 in the eyes of patients with nvAMD may help explain why the majority of patients receiving monthly injections with anti-VEGF therapy have intraretinal and/or subretinal fluid despite treatment (50). The increased expression of ANGPTL4 in nvAMD compared with patients with nnvAMD may further help explain why prophylactic treatment of patients with nnvAMD with anti-VEGF therapy failed to prevent their conversion to nvAMD (65).

Using an in vivo biodegradable nanoparticle-encapsulated siRNA approach, we demonstrate that targeting either ANGPTL4 or VEGF was equally effective in preventing the development of CNV lesions in mice. This suggests that therapies targeting ANGPTL4 may be an alternative approach for the treatment of nvAMD. We further observe that combining therapies targeting both VEGF and ANGPTL4 was more effective for the treatment of CNV in mice than targeting either vasoactive mediator alone. These observations suggest that therapies targeting both ANGPTL4 and VEGF may be a more effective approach for the treatment of CNV.

In this regard, we have recently reported that the endothelial cell receptors NRP1 and NRP2 are essential for the promotion of vascular hyperpermeability by ANGPTL4, similar to VEGF (41). We further observed that ANGPTL4 binds both NRP1 and NRP2 with comparable affinities to VEGFA/NRPs binding. Using sNRP1, which targets both VEGF and ANGPTL4 (41), we were able to effectively inhibit the promotion of endothelial cell tubule formation by ANGPTL4 and VEGF in vitro and CNV lesions in mice.

In addition to ANGPTL4 and VEGFs, NRP receptors have also been reported to bind to other vasoactive mediators, including Class-3 Semaphorins, fibroblast growth factor, platelet-derived growth factor, hepatocyte growth factor, and TGF-β1 (47, 66). sNRPs may, therefore, affect CNV lesions by inhibiting a broad spectrum of vasoactive mediators. While expression of sNRP1 was observed in the eyes of patients with nvAMD, they were not elevated compared with controls. Collectively, these studies provide a foundation for the early clinical assessment of intraocular injections with sNRPs — alone or in combination with current anti-VEGF therapies — in the treatment of patients with nvAMD.

## Methods

### Cell culture and reagents.

iRECs isolated from immortomice were a gift from Jeremy Nathans (Johns Hopkins University School of Medicine, Baltimore, Maryland, USA) and were cultured as previously described (43). HUVECs were cultured as previously described (67). All cell lines were routinely tested for *Mycoplasma* contamination by PCR.

To isolate the primary RPE cells from mice, the eyes from P1–P5 mice were enucleated and digested in 0.2% dispase for 30 minutes on a shaker and then washed in DMEM containing 4.5 g/L of glucose 3 times. The eyes were then dissected to separate the neurosensory retina from the underlying RPE/choroid; the latter was then washed with PBS and then digested in 0.5% trypsin for 45 minutes. The lysate was then washed in DMEM containing 4.5 g/L of glucose and then centrifuged (1000*g* for 5 minutes at room temperature). The supernatant was removed, and DMEM containing 4.5 g/L of glucose was used to resuspend the lysate and then pipetted up and down to physically digest the RPE cells. The lysate was then plated in DMEM containing 4.5 g/L of glucose with 10% FBS.

Aflibercept (Eylea) was purchased from the Johns Hopkins Pharmacy. rhANGPTL4, rmANGPTL4, rhVEGF, and rmVEGF were purchased from R&D Systems. rhsNRP1 was purchased from ReliaTech.

### Quantitative PCR (qPCR).

Primers are listed in Supplemental Table 4.

Total RNA was isolated from culture cells or retinas with PureLink RNA Mini Kit (Invitrogen, 12183025), and cDNA was prepared with MuLV Reverse Transcriptase (Applied Biosystems). qPCR was performed with Power SYBR Green PCR Master Mix (Applied Biosystems) and MyiQ Real-Time PCR Detection System (Bio-Rad). Normalization was done using peptidylprolyl isomerase A (PPIA) for mouse tissue and cell lines.

### Western blot.

Antibodies are listed in Supplemental Table 5.

Cells and RPE/choroid lysates were lysed using radioimmunoprecipitation assay (RIPA) buffer (Sigma-Aldrich) with 10% protease inhibitor cocktail (Cell Signaling Technology). Cell lysates were then solubilized in lithium dodecyl sulfate (LDS) sample buffer (Invitrogen) and incubated for 5 minutes at 95°C. Lysates were subjected to 4%–12% gradient SDS/PAGE (Invitrogen). After blocking with 5% milk (Bio-Rad), the membrane was incubated with primary antibody overnight at 4°C. After washing, the membrane was incubated with HRP-conjugated anti-mouse or anti-rabbit IgG (Cell Signaling Technology 7076/7074) for 1 hour and then visualized with ECL SuperSignal West Femto (Thermo Fisher Scientific). Western blot scans are representative of at least 3 independent experiments.

### In vitro tube formation assay.

iRECs (8000 cells/well) were plated on 10 μL of Matrigel Growth Factor Reduced (GFR) Basement Membrane Matrix, LDEV-free (Corning, 356230), on μ-Angiogenesis slides (Ibidi, 81506) in the presence of the angiogenic factors in 1% serum containing DMEM. The tubes that formed were imaged 16 hours after plating on Cytation 5 imaging multimode reader (Biotek Instruments). Phase contrast images were analyzed for tube length using Angiogenesis Analyzer plugin on ImageJ (Version 1.52a; NIH).

### Mice.

Eight-week-old pathogen-free female C57BL/6 mice were obtained from The Jackson Laboratory. *Hif1a*^+/–^ have been previously described (39). *rho-*h*VEGF* mice (44) were obtained from Peter Campochiaro (Johns Hopkins School of Medicine, Baltimore, Maryland, USA).

### siRNA.

*Hif1a* siRNA*, ANGPTL4* siRNA*, Vegf* siRNA, and a nontargeting control siRNA were purchased from Ambion. Predesigned control (scrambled [scr]), NRP1, and NRP2 siRNA sequences were obtained from Qiagen. siRNA delivery to cells was performed using Hiperfect (Qiagen).

### Intraocular injections.

Intravitreal and subretinal injections were performed with a (PLI-100A) Pico-liter Microinjector (Warner Instruments, Harvard Bioscience) using pulled-glass micropipettes. Each micropipette was calibrated to deliver a 1 μL volume on depression of a foot switch. The mice were anesthetized with a ketamine (100 mg/kg) and xylazine (5 mg/kg) mixture and under a dissecting microscope after pupils were dilated with tropicamide (Alcon Laboratories). The sharpened tip of the micropipette was passed through the sclera just posterior to the limbus into the vitreous cavity and visualized through the dilated pupil. For intravitreal injections, the foot switch was depressed, which caused fluid to penetrate into the vitreous space. For subretinal injections, the tip of the micropipette was advanced to the surface of the neurosensory retina, and the foot switch was depressed, which caused fluid jet to penetrate through the neurosensory retina into the subretinal space.

### FA.

Seventy-two hours after administration of PBS or rmANGPTL4 subretinal injections, 2-week-old *rho-*h*VEGF* mice were anesthetized by a ketamine (100 mg/kg) and xylazine (5 mg/kg) mixture, and pupils were dilated with tropicamide (Alcon Laboratories) and lubricated with hypromellose eye drops (Alcon Laboratories). In total, 5 μL of 10% Sterile Fluorescein Sodium solution (AK-FLUOR 10%) was injected i.p. after the mice were anesthetized. The color fundus photographs and fundus fluorescein angiograms were taken in Micron III of Wilmer core facility.

The FA images were taken in various positions such as central, superior, inferior, temporal, and nasal, and the lesion numbers were counted manually. The pixel size of lesions was converted to mm^2^ by using the following formula. The lesion size (mm^2^) = (fundus area [7.07])/(fundus pixel size [480,000]) × pixel size of the lesion. The quantification area of lesions and fluorescence intensity were done by ImageJ software.

### Laser CNV model.

The laser CNV model in which the laser is used to rupture Bruch’s membrane in mice was performed as previously described (68). Briefly, 10- to 12-week-old mice were anesthetized with a mixture of ketamine (100 mg/kg) and xylazine (5 mg/kg) after pupils were dilated with tropicamide (Alcon Laboratories). Lubricating hypromellose eye drops (Alcon Laboratories) were applied to the cornea. The fundus was viewed using a hand-held coverslip as a contact lens, and laser photocoagulation was performed using the diode laser photocoagulator (IRIS Medical) and the slit lamp delivery system. Three to 4 laser burn spots equidistant from the optic nerve using a wavelength of 532 nm, a power of 160–190 mW, a duration of 100 ms, and a spot size of 75 μm were performed for each eye. Treatments (when indicated) provided on day 3, including aflibercept (800 ng, PBS with equal volume as vehicle control) or nanoparticle RNAi were introduced by intravitreal injection as described above. Animals were sacrificed at day 7, eyes were enucleated, and further studies were conducted for immunofluorescence of laser CNV lesions (described below).

### In situ hybridization.

RNA in situ hybridization was performed with RNAscope 2.5 HD Duplex Detection Reagent Kit (ACD, 323350) following the manufacturer’s protocol. Fresh eyecups, without prior fixation, were embedded into OCT compound (Tissue-Tek) and immediately frozen by liquid nitrogen. The frozen blocks were sectioned at a thickness of 14 μm and were assayed for mouse *Vegf* (probe no. 405131) or human *VEGF* mRNA (probe no. 423161) versus negative control (probe no. 320751). The signal was visualized and captured by a Zeiss confocal microscope meta 710 LSM (Carl Zeiss Inc.).

### IHC.

Immunohistochemical detection was performed according to the manufacturer’s protocol on cryopreserved human tissue sections (obtained from the Wilmer Eye Institute Ocular Pathology Archives with approval from the Johns Hopkins School of Medicine IRB) by using a nitroblue tetrazolium development system as previously described (69, 70) according to the manufacturer’s protocol on nvAMD disease patients. Images were captured by scanning slides using the Aperio ScanScope program on Aperio Scanscope XT System (Leica Biosystems).

### Immunoﬂuorescence.

Antibodies and dilutions are listed in Supplemental Table 5.

The immunoﬂuorescence for the laser CNV model was performed on choroidal flat mounts, and cross-sections were performed as previously described (37). For choroidal flat mounts, eyes were enucleated and fixed in 4% paraformaldehyde solution (Thermo Fisher Scientific) in PBS for 2 hours at room temperature (RT) or overnight at 4°C followed by washing with PBS 3 times for 10 minutes in a shaker. RPE/choroid was isolated and incubated in a 0.5% BSA solution overnight at 4°C. They were then washed with PBS 3 times for 10 minutes in a shaker. HypoxyProbe was incubated with the flat-mount retina at the indicated time points. Isolectin B4 (Invitrogen) or CD31 was used to label the choroidal vasculature and incubated overnight in 4°C. After 3 washes with PBS for 10 minutes each in a shaker, choroidal flat mounts were prepared. Images were captured at high magnification (20×) with Zeiss fluorescent microscope (Carl Zeiss Inc.), and the area of CNV lesions was calculated as mm^2^ by ImageJ software by keeping the parameters the same for all the spots.

For cross sections, eyes were fixed in 4% paraformaldehyde for 2 hours. After washing in PBS, the tissues were embedded in OCT containing 6% agarose (w/v), and 10 μm–thick slices were cut. Cross-sections were taken through laser-induced CNV lesions and stained with CD31; labeling of retinal vasculature was used as an anatomical landmark for the presence or absence or vessels.

For double-labeling, samples were incubated in a blocking solution with 5% normal donkey serum plus 0.3% Triton X-100 in saline for 1 hour at RT. Subsequently, the samples were incubated in a mixture of primary antibodies overnight at 4°C. After washing, the slices were incubated in secondary antibodies for 1 hour at RT.

Immunoﬂuorescence was performed using donkey anti–mouse Alexa Fluor 488/555 (Invitrogen, A-21202/31570), donkey anti–goat Alexa Fluor 647 (Invitrogen, A-21447), or donkey anti–rabbit Alexa Fluor 488/555 (Invitrogen, A-21206/37118), in combination with DAPI (Invitrogen). Images were captured by the LSM 710 confocal microscope (Carl Zeiss Inc.) or the EVOS FL Auto 2 Imaging System (Thermo Fisher Scientific).

### Nanoparticles.

rBEAQs were synthesized as previously described (46). Briefly, bioreducible monomer 2,2-disulfanediylbis(ethane-2,1-diyl) diacrylate (BR6), diacrylate monomer bisphenol A glycerolate (1 glycerol/phenol) diacrylate (B7), tri-acrylate monomer trimethylolpropane triacrylate (B8), and side chain monomer 4-amino-1-butanol (S4) were reacted overnight at 90°C with stirring to form the acrylate-terminated base polymer, which was then end-capped with 2-(3-aminopropylamino)ethanol (E6) to form the final polymer used in these studies (Figure 7A and Supplemental Figure 2). siRNA-encapsulating nanoparticles were formed by dissolving siRNA and polymer at the desired concentrations in 25 mM sodium acetate solution (NaAc; pH 5) and mixing the 2 solutions at a 1:1 volume ratio. Nanoparticles were allowed to self-assemble for 10 minutes at RT, at which time they were mixed at a 1:1 volume ratio with sodium bicarbonate solution at a final concentration of 9 mg/mL (NaHCO_3_; pH 9). For in vitro transfections, nanoparticle solutions were added directly to cell culture media and incubated for 4 hours. Nanoparticles were formulated at a polymer: siRNA weight (WT/WT) ratio of 40; the final siRNA dose was 100 nM per well. For transfections simultaneously knocking down VEGF and ANGPTL4, siRNA targeting each sequence was premixed at a 1:1 molar ratio prior to mixing with polymers; siRNA dose in this case was 50 nM per well, per siRNA sequence.

For intravitreal injections, nanoparticles were synthesized at a polymer concentration of 10 mg/mL to enable a higher dose to be delivered in the limited injection volume. Nanoparticles were lyophilized in the presence of sucrose (30 mg/mL) as a cryoprotectant and resuspended using deionized water to a final isotonic sucrose concentration of 100 mg/mL immediately prior to injection.

### Patient samples.

Aqueous samples were collected from consenting patients with nvAMD at the Wilmer Eye Institute undergoing cataract and/or vitrectomy surgery or an intravitreal injection. Control samples were obtained from patients without nvAMD, diabetes mellitus, or retinal disease at the time of cataract surgery. At the time of intravitreal injection or cataract and/or vitrectomy surgery, aqueous fluid was collected via limbal paracentesis using a 30-gauge needle attached to a tuberculin syringe. Aqueous samples were immediately processed and stored at –80°C prior to analysis.

### ELISA.

ANGPTL4 (DuoSet, catalog DY3485), VEGF (Quantikine, catalog DVE00), ANGPT2 (Quantikine, catalog DANG20), and sNRP1 (Quantikine, catalog DNRP10) ELISA kits were purchased from R&D Systems. Aqueous fluid samples were diluted 1:10 and analyzed for ANGPTL4, VEGF, ANGPT2, or sNRP1 protein according to the manufacturer’s protocols.

### Statistics.

In all nonclinical studies, data are shown as a mean ± SD from at least 3 independent experiments. Data from clinical samples are shown as mean ± SD. ROC curves for each respective vasoactive mediator were generated using MATLAB. Statistical differences between groups was performed with Prism 8.0 software (GraphPad) and were determined by Kruskal-Wallis and Dunn’s or Dunnett’s T3 multiple-comparison tests, Wilcoxon rank sum test, 2 tailed Student’s *t* test, 1-way ANOVA with Bonferroni correction, χ^2^ test, and Spearman correlation as indicated. Statistical significance was defined as *P* less than 0.05.

### Study approval.

All animals were treated in accordance with the Association for Research in Vision and Ophthalmology Statement for the Use of Animals in Ophthalmic and Vision Research and the guidelines of the Johns Hopkins University Animal Care and Use Committee. IRB approval (NA_00075565) from the Johns Hopkins University School of Medicine, and informed consent was obtained for all patient samples used in this HIPAA-compliant study.

## Author contributions

AS and SM are the primary contributors to research design. YQ, AD, KJ, MR, XC, JCS, CG, JZ, JV, DM, NSK, TM, SYT, and YD are responsible for research execution and are contributors to data acquisition. AS, YQ, AD, KJ, MR, XC, JCS, CG, JZ, DM, TM, SYT, JJG, and SM are the primary contributors to data analysis and interpretation. Manuscript preparation was contributed by AS and SM, with revisions provided by YQ and GLS.

## Supplementary Material

Supplemental data

## Version 1 06/02/2022

In-Press Preview

## Version 2 07/08/2022

Electronic publication
