# Supplementary material for: ANGPTL4 influences the therapeutic response of patients with neovascular age-related macular degeneration by promoting choroidal neovascularization
Source: JCI Insight. 2022 Jul 8;7(13):e157896. doi: 10.1172/jci.insight.157896 (PMC9310537; doi:10.1172/jci.insight.157896)

**Supplemental Figure Legends**

**Supplemental Figure 1. Expression of ANGPTL4 in patients with nvAMD.**

(**A** and **B**) Aqueous levels of ANGPTL4 in treatment-naïve patients with active nvAMD (i.e., nvAMD patients who have never received anti-VEGF therapy; nvAMD UnTx; A) or patients with nvAMD previously treated with anti-VEGF therapy 12 or more weeks prior to sample collection (nvAMD Recurrent; B) compared to patients with nvAMD and non-AMD (Control) patients. NOTE: Red circle identifies patient sample not displayed in Figure 3B to adequately demonstrate the variability within the nvAMD samples. Kruskal-Wallis with Dunn's multiple comparisons test, *$P < 0.05$; **$P < 0.01$; ***$P < 0.001$; ****$P < 0.0001$. NS, not significant.

**Supplemental Figure 2. Chemical structure of rBEAQ polymer for *in vivo* delivery of siRNA.**

Chemical structures for R groups of rBEAQ polymer.

## Supplemental Tables

**Supplemental Table 1.** Baseline characteristics of TEP/M and Control patients whose aqueous samples were used for ELISAs.

| Characteristic | q4 (n=10) | q6-8 (n=9) | q10-12 (n=6) | Weaned (n=13) | Control (n=39) |
|---|---|---|---|---|---|
| Mean Age (yr) | 76.2 ± 1.6 | 78.6 ± 3.0 | 81.2 ± 1.9 | 82.5 ± 2.4 | 70.0 ± 1.5 |
| Female % (#) | 60% (6) | 56% (5) | 83% (5) | 77% (10) | 56% (22) |
| Pseudophakic % (#) | 90% (9) | 89% (8) | 33% (2) | 85% (11) | 23% (9) |

Abbreviation: n, sample size; yr, years; q4, TEP/M patients who required treatment with anti-VEGF therapy every 4 weeks at the end of their first year of treatment; q6-8, TEP/M patients who required treatment with anti-VEGF therapy every 6-8 weeks at the end of their first year of treatment; q10-12, TEP/M patients who required treatment with anti-VEGF therapy every 10-12 weeks at the end of their first year of treatment weeks; Weaned, TEP/M patients who were weaned off treatment with anti-VEGF therapy for 30 weeks or longer at the end of their first year of treatment; and ELISA, enzyme-linked immunoassay. Values displayed as mean ± standard error of the mean.

**Supplemental Table 2.** Performance characteristics of each angiogenic mediator alone and in combination when used to predict patients who were likely to require continuous monthly treatment vs those who required intermittent treatment and those who were able to be weaned.

| **Angiogenic Factor** | **Sensitivity** | **Specificity** | **AUC** |
|---|---|---|---|
| VEGF | 0.833 | 0.529 | 0.696 |
| ANGPTL4 | 0.909 | 0.679 | 0.802 |
| ANGPT2 | 0.714 | 0.454 | 0.513 |
| VEGF + ANGPT2 | 0.595 | 0.743 | - |
| VEGF + ANGPTL4 | 0.758 | 0.849 | - |
| ANGPTL4 + ANGPT2 | 0.649 | 0.825 | - |

Abbreviations: AUC, area under curve; VEGF, vascular endothelial growth factor; ANGPTL4, angiopoietin-like 4; ANGPT2, angiopoietin 2.

**Supplemental Table 3.** Patient Characteristics for aqueous samples.

| Patient Characteristic | Control (N=38) | nnvAMD (N=12) | nvAMD Untreated (N=20) | nvAMD Recurrent (N=20) | nvAMD First Treatment§ (N=12) | *P* |
|---|---|---|---|---|---|---|
| Mean Age in Years ± SD | 65.6 ± 11.8 | 76.1 ± 5.7 | 81.0 ± 5.5 | 80.6 ± 10.6 | 78.1 ± 6.6 | <0.0001 |
| Males – no. (%) | 18 (47) | 5 (42) | 5 (25) | 5 (25) | 6 (50) | 0.28 |
| Pseudophakic – no. (%) | 0 (0) | 0 (0) | 16 (80) | 13 (65) | 7 (58) | <0.0001 |
| Prior vitrectomy – no. (%) | 0 (0) | 0 (0) | 0 (0) | 0 (0) | 0 (0) | – |
| DM – no. (%) | 0 (0) | 3 (25) | 5 (25) | 4 (20) | 5 (42) | 0.005 |
| CVD[a] – no. (%) | 27 (71) | 7 (58) | 17 (85) | 19 (95) | 10 (83) | 0.09 |

nnvAMD, non-neovascular age-related macular degeneration. nvAMD, neovascular age-related macular degeneration. DM, diabetes mellitus. CVD, cardiovascular disease.
§Includes unique samples from the same eyes of 8 patients from the nvAMD untreated group.
[a]Includes any patient with a history of hypertension, hypercholesterolemia, coronary artery disease, or cerebral vascular accident.

**Supplemental Table 4.** Primer sequences for Real-Time (RT)-PCR

| | Gene | | Sequence (5'to 3') |
|---|---|---|---|
| Mouse | | | |
| | *Angptl4* | Forward | TTGGTACCTGTAGCCATTCC |
| | | Reverse | GAGGCTAAGAGGCTGCTGTA |
| | *Vegf* | Forward | CTTATACAGGAATGGAGGCTGT |
| | | Reverse | TTCACCTGACAGGATTGGATAAT |
| | *PPIA* | Forward | AGCATACAGGTCCTGGCATC |
| | | Reverse | TTCACCTTCCCAAAGACCAC |
| | *Hif1a* | Forward | GCACGGGCCATATTCATGTC |
| | | Reverse | CACGTCATGGGTGGTTTCTTG |

**Supplemental Table 5.** Antibodies used in this study.

| Name | Company | Species | Catalog Number | dilution | Marker or Applications |
|---|---|---|---|---|---|
| HIF-1α | Gene Tex | rabbit | GTX127309 | 1:200 or 1:1000 | WB |
| HIF-1α | Novus | mouse | NB-100-105 | 1:500 | IF |
| HIF-1α | abcam | rabbit | Ab2185 | 1:500 | IHC |
| β-Actin | Cell signaling | rabbit | 4967 | 1:5000 | WB |
| GAPDH | sigma | mouse | G8795 | 1:10000 | WB |
| Isolectin GS IB4 | Thermo Fisher | | I21413 | 1:200 | IF |
| CD31 | R&D | goat | AF3628 | 1:1000 | IF |
| VEGF | Santa Cruz | rabbit | Sc-152 | 1:1000 | IHC/IF |
| ANGPTL4 | abcam | rabbit | Ab115798 | 1:400 | IHC/IF |
| Nrp1 | abcam | Rabbit | Ab81321 | 1:1000 | WB |
| Nrp2 | Santa Cruz | Mouse | Sc13117 | 1:1000 | WB |
| 2° antibodies | Invitrogen | | | 1:1000 | IF |
| 2° antibodies | Dako | | | 1:100 | IHC |

Supplemental Figure 1

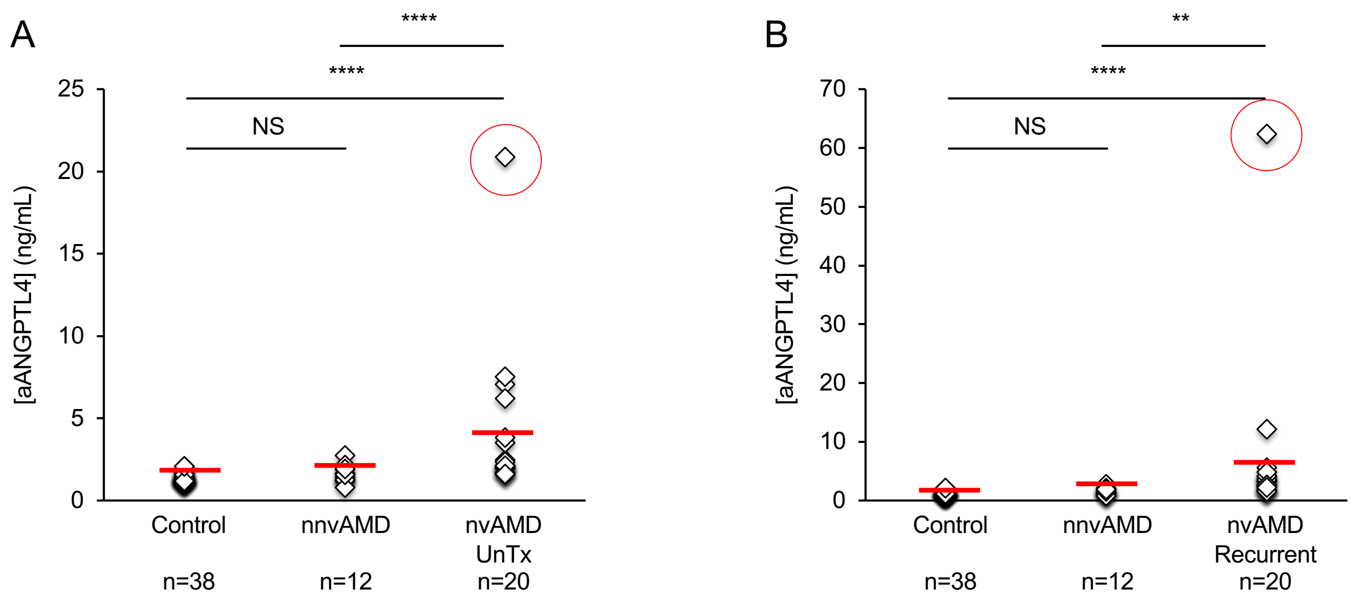

Supplemental Figure 2

BR6

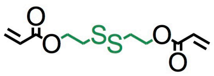

B7

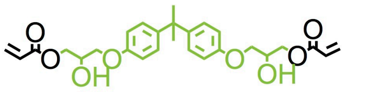

B8

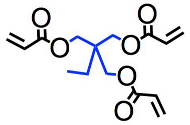

S4

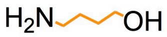

E6

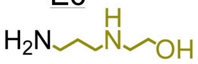

rBEAQ

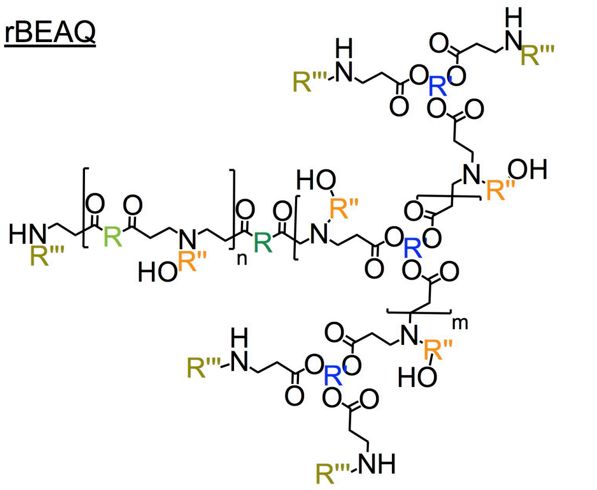

Supplement: Supplemental data [file jciinsight-7-157896-s160.pdf]
